# Hysteretic Swelling/Deswelling of Polyelectrolyte Brushes and Bilayer Films in Response to Changes in pH and Salt Concentration

**DOI:** 10.3390/polym13050812

**Published:** 2021-03-06

**Authors:** Nisha Hollingsworth, Ronald G. Larson

**Affiliations:** 1 Department of Macromolecular Science & Engineering, University of Michigan, Ann Arbor, MI 48109, USA; nrhollin@umich.edu; 2 Department of Chemical Engineering, University of Michigan, Ann Arbor, MI 48109, USA; 3 Biointerfaces Institute, University of Michigan, Ann Arbor, MI 48109, USA

**Keywords:** polyelectrolytes, brush, hysteresis, quartz crystal microbalance

## Abstract

We use a quartz crystal microbalance with dissipation (QCM-D) to investigate the swelling/de-swelling and hysteresis in brushes of weakly ionizable polyanion poly(acrylic acid) (PAA) brushes and bilayers containing a PAA brush and a poly(ethylene imine) (PEI) overlayer. We show that for a long PAA chain (M_w_ = 39 kDa), at low grafting density (σ < 0.05 chains/nm^2^) and at a pH value (=4.2) at which it is partially charged, in the low-salt “osmotic brush” regime, the brush height no longer increases with increased grafting density as is seen for shorter brushes and denser grafting, but shows a slight decrease in height, in qualitative agreement with predictions of scaling theory. In a cycle of stepped pH changes, we also show that at a low grafting density of σ = 0.023 chains/nm^2^ and M_w_ = 39 kDa, there is hysteresis in swelling over timescales of many minutes. For higher grafting densities σ = 0.87 chains/nm^2^ and shorter chains (2 kDa), we see little or no measurable hysteresis, and, at intermediate chain length 14 kDa and grafting density σ = 0.06 chains/nm^2^, hysteresis is observed at short timescales but is greatly reduced at longer timescales. These results are similarly observed when bilayers are made by adsorbing onto the PAA brush a layer of the polycation PEI. In addition, we also note hysteresis in swelling upon changes of salt concentration when pH is fixed. These results show the rich thermodynamics and kinetics of even monolayers and bilayers of polyelectrolyte films.

## 1. Introduction

Polyelectrolyte films, including adsorbed monolayers, or “brushes,” as well as multilayers, made for example by layer-by-layer (LbL) assembly, have continued to gain popularity in recent years due to their applications in areas ranging from waterborne lubrication and colloidal stabilization to biomaterials [1,2,3,4]. Weak polyelectrolyte brushes are particularly desirable because their extent of charge varies with pH, making them pH responsive. LbL films have also gained popularity because of their potential applications in electronics and drug delivery [5,6,7]. LbL multilayers are formed by the sequential adsorption of polyelectrolytes whose charge alternates in sign from one layer to the next, resulting in films ranging from nanometers to microns in thickness, with great flexibility in layer composition. This is especially useful in a field such as drug delivery, where individual film layers may be loaded with small molecule drugs to deliver a specific profile of release versus time.

While their possible applications are many, much is left to be understood about polyelectrolyte (PE) films, including non-equilibrium phenomena during film growth or after a change in pH, salinity, or temperature. Molecular simulations and theoretical models have been developed for the growth of LbL films [8,9,10,11], but these do not fully describe experimental results. Indeed, LbL film growth depends critically on non-equilibrium behavior, since attainment of true equilibrium would imply the rinsing away of previously deposited layers. While previous theoretical work has invoked a “sequential equilibrium” approximation [12] in which previously deposited layers are assumed to be “frozen” in place upon subsequent deposition steps, more realistic treatments of LbL dynamics that allow for partial desorption of previously adsorbed layers, are only just beginning. The recent theory of Salehi and Larson [11], for example, allows for partial desorption during rinsing, but has only been applied to bilayers, not full LbL films.

The complexity of LbL growth leads us to focus attention on the simpler cases of a grafted polyelectrolyte monolayer and to a bilayer produced by adsorption of a second, oppositely charged, polyelectrolyte to the grafted monolayer. While such systems differ significantly from LbL multilayers, understanding even these simpler systems is a challenge that likely must be overcome before one can hope to develop a quantitative theory for LbL multilayer deposition. For example, the accuracy of a recent theory of LbL film growth by Salehi and Larson [11] has yet to be confirmed experimentally even for a bilayer. Moreover, polyelectrolyte brushes and bilayers are of interest in their own right, and if they are to be used in environments with changing pH or salinity, as is often the case especially for biological or sensing applications, it is important that their non-equilibrium response be well-understood.

Here, we focus on measuring hysteresis in response to changes in pH and salinity in “simple” weakly ionizable PE brushes, and in bilayers formed by adsorption of an oppositely charged weak PE onto the brush. We focus on brushes as a starting layer, since their deposition is much better controlled than are the adsorbed layers often used to initiate LbL multi-layer deposition. Moreover, future studies of LbL deposition might benefit by initiating growth of the multi-layer from a brush rather than an adsorbed layer, since the properties of the adsorbed layer are highly sensitive to surface preparation, and subsequent multilayer growth is affected by how well and how densely the first layer is attached to the surface. Initiating the multilayer with a well-defined brush should avoid these complications.

Our previous work focused on the swelling of brushes of poly(acrylic acid) (PAA) at various fixed pH values, chain lengths, salt concentrations, and grafting densities. There, we used a quartz crystal microbalance with dissipation (QCM-D) to measure the steady-state swelling of a weakly dissociating brush of poly(acrylic acid) (PAA), which, via a thiol-gold linkage, had been self-assembled as a monolayer onto a gold-coated silica QCM-D crystal. QCM-D is a well-known instrument for measuring subtle changes in adsorbed mass and viscoelasticity in both polyelectrolyte brushes and LbL films in real time, allowing the swelling dynamics to be tracked accurately [13,14,15].

The grafting density of the resultant brushes was controlled reproducibly through use of a competitive adsorber and through variations in solution concentration [16]. The measured dependencies studied (on salt concentration, chain length, and grafting density at three pH values) allowed confirmation of the scaling theory for weak polyelectrolytes [17] except for a failure to find the predicted inverse relationship between brush height and grafting density in the low-salt “osmotic brush” regime. In addition, independent experiments using a freshly prepared brush were used for each condition, so that possible hysteresis arising from forward and reverse changes in pH or salt concentration within a single sample was not considered, and thus not observed. Hysteretic swelling in response to changes in pH has previously been reported in both LbL films of PAA and Poly(allylamine hydrocholoride) (PAH) [18] and brushes of PAA [19,20], and theory has suggested the possibility of hysteresis in response to changes in polyelectrolyte concentration or grafting density [21]. Thus, it is of interest to extend our work to see if our brushes also show hysteresis under at least some conditions. In addition, our previous work considered only a monolayer, and extending the work to a bilayer formed by adsorbing an oppositely charged polyelectrolyte to the monolayer would be an obvious next step toward a longer-term goal of understanding the behavior of a multi-layer.

Thus, the above-mentioned three limitations of our previous work: i.e., (1) incomplete validation of monolayer scaling theory, (2) lack of information on hysteresis or slow kinetics of monolayer response to changes in salt or pH, and (3) the lack of presence of a second, oppositely charged polyelectrolyte, all motivated the new work presented here.

Here, we again use QCM-D, this time to measure the swelling kinetics of PAA brushes, with and without an adsorbed complementary weak polycation, poly(ethylene imine) (PEI), to form a bilayer. First, we revisit the salt-dependent swelling of a long-chain brush of PAA at lower grafting densities and pH values close to the pKa (~5) of PAA in an effort to reconcile results with the brush-thickness scaling laws predicted in the theory of Zhulina et al. for weakly charged polyelectrolyte brushes in the osmotic brush regime [17]. Then, we investigate the time-dependent swelling responses of these brushes, and the bilayers made from them by adsorbing PEI, to consecutive upward and downward changes in pH. The ability to measure the time-dependence of the swelling response is an advantage of using QCM-D that heretofore had not been taken advantage of.

## 2. Materials and Methods

Materials. We prepared all brush samples as discussed in prior work [16]. We obtained three α-thiol ω-bromo terminated poly(acrylic acid) (PAA-SH) samples (short-chain: M_n_ = 2000 g/mol, M_w_ = 2600 g/mol, M_w_/M_n_: 1.3, –SH > 99%; medium-chain: Mn = 14,000 g/mol, Mw = 18,200 g/mol, Mw/Mn: 1.27, –SH > 99%; long-chain: M_n_ = 39,000 g/mol, M_w_ = 55,000 g/mol, M_w_/M_n_: 1.4, –SH > 99%) from Polymer Source Inc. (Quebec, Canada). We obtained linear polyethyleneimines (PEI) (3 chain lengths: M_n_ = 2500 g/mol, M_n_ = 10,000 g/mol, M_w_ = 25,000 g/mol, M_w_/M_n_ < 1.3) from Polysciences, Inc. (Warrington, PA, USA) and Millipore Sigma (St. Louis, MO, USA). Potassium chloride (KCl) obtained from Millipore Sigma USA was used as received. HPLC plus grade water (pH 7) (Millipore Sigma, St. Louis, MO, USA) was used to prepare all salt solutions, at concentrations between 0.1 and 1000 mM. Solutions of KCl were found to exhibit pH 7 upon mixing without buffer; we obtained pH values of 1–9 in these solutions upon the addition of different concentrations of hydrochloric acid (HCl) (Sigma Aldrich, St. Louis, MO, USA) or potassium hydroxide (KOH) (Sigma Aldrich, St. Louis, MO, USA), respectively, all with concentrations <3 mM. The pH and ionic strengths were confirmed using both a conductivity meter (CON 6+, Oakton) and a benchtop pH meter (Accumet AE150, Fisher Scientific, Waltham, MA, USA) calibrated against reference standards (The pH values reported in what follows are those of stock solutions introduced into the QCM-D flow chamber. It is possible that actual pH values of solutions in direct contact with the brushes might vary due to ion exchange).

Preparation of gold-coated QCM-D crystals. We purchased gold-coated silicon wafers (QSX 301, AT-cut, *F*_0_/*n* = 5 MHz) from Q-Sense by Biolin Scientific (purchased through supplier Nanoscience Instruments, Phoenix, AZ, USA). Prior to deposition of PAA, we cleaned the sensors via a TL-1 base cleaning solution, deionized water, absolute ethanol, and O_2_- plasma as described previously [16].

Preparation of PAA-SH brushes. Gold-coated silicon wafers were plasma treated before use. As described previously [19], we dissolved short-chain PAA-SH (M_n_: 2000 g/mol, M_w_/M_n_ = 1.3) in absolute ethanol to a final concentration of 4 mM [22]. The 4 mM PAA solution was used both as is and after dilution in either absolute ethanol or a 10:1 (wt%) mixture of absolute ethanol to 2-mercaptoethanol, where the latter is a competitive adsorbent to compete with the PAA-SH in binding to the gold-coated QCM-D crystal, thereby controlling grafting density. Then, we deposited 40 μL of the PAA solution onto the crystal, incubated it for 18 h, and rinsed repeatedly with absolute ethanol and dried, prior to QCM-D analysis. Preparation of medium-chain PAA-SH (M_n_: 14,000 g/mol, M_w_/M_n_ = 1.27) was similar to that for short chains, except the incubation was for only 60 min, rather than the 18 h used for the short brush, since incubation longer than this led to the formation of a thick, heterogenous physical film due presumably to solvent evaporation. For the long-chain PAA-SH (M_n_: 39,000 g/mol, M_w_/M_n_ = 1.4), incubation was for 20 min. The widely varying incubation times were needed to control grafting density, as described in detail can in earlier work [19].

Variable Angle Spectroscopic Ellipsometry (VASE). We obtained the thickness of the dry PAA-SH brush on the gold-coated QCM-D crystal by variable angle spectroscopic ellipsometry (VASE) (Woollam M-2000 Spectroscopic Ellipsometer, J. A. Woollam, Linocln, NE, USA) as described previously [16], using the parameters recommended for a PAA layer on bulk gold by Bittrich et al. [23] and the angles of incidence and fitting wavelengths used in our previous work [16]. As before, we dried samples thoroughly in vacuum prior to VASE measurement to remove all water. The optical relative phase shift, Δ, and relative amplitude ratio, tan (Ψ), measured by VASE, were modeled using two slabs, as described before [19], taking the PAA layer to be transparent (k_film_(λ) = 0) and homogenous, and with index of refraction (*n*_PAA_) of 1.522 at λ = 631.5 nm. We found its optical thicknesses from measured values of Δ and Ψ using conventional methods [24,25]. See previous work for details [19].

Quartz Crystal Microbalance with Dissipation (QCM-D). We used the same Q-Sense E4 system (Q-Sense AB, Gothenburg, Sweden) as previously [19]. Initially, we established fundamental frequency signals on the prepared sensors in air. We pumped solutions through the device at a constant flow rate of 0.100 mL/min at room temperature (~22 °C). We conducted experiments investigating the scaling of brush height with respect to grafting density as previously described [16], where for each grafting density, each result for a given salinity was obtained for a newly deposited brush, solvated with the desired buffer, rather than using step-wise changes in buffer conditions applied to the same brush, as done for the hysteresis studies. As before, we performed experiments in triplicate (N = 3) and error bars represent standard deviation, which for these studies, are smaller than the symbols.

In the case of the pH hysteresis experiments for a single brush layer, we established a baseline for each measurement by flowing ultra-pure water (pH 7) until a steady response was seen, followed by the appropriate KCl solution buffered in a succession of steps in pH (pH 7-4-1-4-7-9) with a pre-determined time interval at each pH, typically 4 min, although in some cases longer, as discussed in the Results section. For the experiments involving a PAA brush layer with an adsorbed overlayer (i.e., a bilayer), the complementary polyelectrolyte PEI was introduced to the adsorbed PAA brush under flow until a steady-state was achieved, followed by a rinse step (with no polymer, at the appropriate pH and KCl concentration), again, allowing a steady-state to be reached. We then subjected this bilayer steady-state baseline measurement to the same pH steps as described previously for a brush layer alone, again with pre-determined time intervals at each pH. After each change in pH was introduced at the inlet, it took around six minutes for this change to be registered in a step-like response of the QCM-D signal. Once the response to the pH change was observed in the signal, we averaged the height over the entire time interval between the jumps in response, irrespective of any time dependence in the signal over this period. In some cases, most or all of the signal over which averaging occurred was time dependent, as described in the Results and Discussion section. Since the time-dependence is highly reproducible and sensitive to the brush and salt and pH change, we believe that this time dependence represents the kinetics of the response of the brush to the change in pH, and not instrumental lag or baseline drift. Experiments were performed in triplicate (N = 3) and time averages over each run were then themselves averaged over the three runs and the standard deviation for these three is reported as the error bar.

We used the change in frequency Δ*F* to calculate the change in mass using the Sauerbrey relation rather than a viscoelastic model for the reasons described previously [16]. Again, the work of DeNolf et al. [26] demonstrated that the Sauerbrey relation holds when the film is thin and/or rigid enough; specifically, *d*/λ*_n_*, the ratio of the thickness of the film *d* to the shear wavelength of the material λ_n_, must be no greater than 0.05 [26,27], a condition met in all of the brushes and bilayers studied here.

As mentioned previously, the Sauerbrey relation yields a quantitative measure of the adsorbed mass and swelling through the measurement of Δ*F* [27]. Details of the set-up of the QCM-D experiment are described in previous work [16]. Only one overtone (*n* = 3) is used to interpret the Sauerbrey mass [28]. The mass change (Δ*m*) calculated using Sauerbrey relation for rigid films is given by Equations (1) and (2) [29].

(1) $$\Delta F=-\Delta m\left(\frac{2nF_{0}^{2}}{A\sqrt{\mu_{q}\rho_{q}}}\right)=-\frac{1}{C_{f}}\Delta m$$

(2) $$\Delta d=\frac{\Delta m}{\rho }$$

We obtained the crystal parameter (*C_f_*) from the following parameters given by the manufacturer: *F*_0_ is the fundamental frequency (=5 MHz), *n* the overtone number (=3), Δ*m* the change in mass per unit area, *ρ_q_* the density of quartz (=2.648 g/cm^3^), A the crystal area (=1.54 cm^2^), and *μ_q_* is the shear modulus of quartz (=2.947 × 10^11^ g·cm^−1^·s^−2^), respectively [13,30,31]. We converted the Sauerbrey mass difference Δm into a thickness increment Δd using the density *ρ* of the added-mass. A more negative Δ*F* indicates that more mass is adsorbed.

As before [19], we obtain adsorbed brush mass by ellipsometry and use QCM-D to obtain changes in brush mass due to solvation of the brush. In our previous work, we demonstrated the importance of correcting QCM-D data obtained with a polymer brush by subtracting from these data the results obtained for a solvent-covered polymer-free crystal. This subtraction removes the effects of the density and viscosity of the solvent, which vary with salt concentration. To obtain our results, we assume the same additive relationship between the effects of the polymer brush described in previous work [16] and in great detail by Chu et al. [32].

## 3. Results

### 3.1. Variable Angle Spectroscopic Ellipsometry (VASE)

We measured the dry thicknesses of PAA brushes and PAA/PEI bilayers by variable angle spectroscopic ellipsometry (VASE). The grafting density *σ* in chains/nm^2^ was calculated using the same method and formula as described in previous work [16]. These findings are summarized in Table 1.

### 3.2. Scaling Theory for Monolayer PE Brushes and Comparison to Previous Findings

Zhulina et al. [20] predict that in the osmotic brush (OB) regime corresponding to an extremely low external ionic strength, the height H of a weakly dissociating polyelectrolyte brush should scale according to Equation (3) with respect to the number of monomers in the chain *N*, the grafting density σ, the ionic strength given as the sum of the external proton concentration [*H*^+^], and the salt concentration (*C_S_*).

(3) $$H\sim N\sigma^{-\frac{1}{3}}{\left(\left[H^{+}\right]+C_{s}\right)}^{1/3}$$

Zhulina et al. [20] also predicted that in the salted brush (SB) regime corresponding to an external salt concentration above the critical value, the brush height H should scale according to Equation (4).

(4) $$H\sim N\sigma^{\frac{1}{3}}C_{s}{{}^{-}}^{\frac{1}{3}}$$

By comparing Equations (3) and (4), we see that while the scaling of brush height with chain length is the same in the osmotic and salted brush regimes, the scaling with grafting density and salt concentration are inverted in the osmotic brush regime, relative to the salted brush regime. In our previous work, the same PAA brushes as used in this work were studied to test these scaling relationships at multiple fixed pH values at various grafting densities across a broad range of salt concentrations [16]. It was found that, as predicted by Equations (3) and (4), when ionized, the brushes initially swell at low external salt (<10 mM), corresponding to the low ionic strength OB regime, and de-swell at higher external salt concentrations, in the SB regime, giving very similar results at the two fixed pH values of 7 and 9. At pH 3, where the brushes are expected to be charge neutral, their behavior was found to be drastically different; they no longer exhibit either of these swelling regimes and instead are nearly unaffected by the external salt concentration [16].

To explore more carefully the transition from the behavior at pH 7 and 9, and that at pH 3, it is of interest to examine their behavior at pH values below, but closer to, the pKa (~5). Such an investigation is especially of interest, since our previous findings for PAA brushes agreed with the scaling dependences on salt concentration predicted by Zhulina et al. in both osmotic and salted regime, but agreed with the predicted dependence on grafting density only in the salted brush regime [16]. In the osmotic brush regime, our earlier work showed a brush height roughly proportional to the +1/3 power of grafting density σ, similar to that in the salted brush regime, but disagreeing with Equation (3). However, in our previous work the effect of grafting density was studied only at relatively high grafting density and only for the shortest brushes (2 kDa), which were highly stretched, deviating from the assumption of ideal Gaussian elasticity in the theory of Zhulina et al. [17]. Our previous findings might therefore disagree with scaling predictions Zhulina et al. both because our chains were not weakly ionized at the pH values considered, and also because they were highly stretched and not close to the region of Gaussian elasticity.

**Figure 1:**
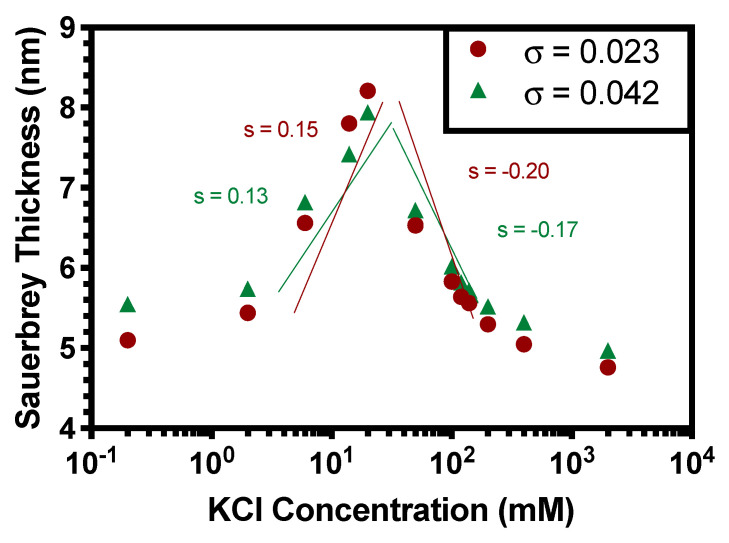
Height (i.e., Sauerbrey thickness) at various salt concentrations for a 39 kDa PAA brush at grafting density σ = 0.023 chains/nm^2^ (red circles) and σ = 0.042 chains/nm^2^ (green triangles) at pH 4.2, below the pKa (~5). Log-log fits demonstrate the scaling exponents s for the Osmotic Brush (OB) regime (left slopes) and Salted Brush (SB) regimes (right slopes).

In the present study, these limitations are rectified by using our long-chain PAA brush which we diluted to a low grafting density σ of 0.023 chains/nm^2^, which is significantly more sparsely grafted than for the short-chain brush in this work and for the long-chain brush in our previous experimental work [16] (σ = 0.45 chains/nm^2^). Thus, our new, longer, more sparsely grafted chains are closer to the Gaussian regime. We will show that for these chains the brush collapses partially at pH 4, while at pH 1 they can be considered to be fully charge neutral. Guided by this transition at around pH 4, Figure 1 demonstrates the swelling response of this same brush at fixed pH = 4.2, where it is presumably partially ionized, with respect to variations in KCl concentration. For this brush at this pH, the charge fraction is considerably lower than unity and the brush can be considered to be partially ionizable, rather than more nearly fully ionized, as expected at the pH values investigated previously (pH 7 and 9) [16]. Furthermore, the brushes investigated are orders of magnitude more sparsely grafted than previously, since σ = 0.023 chains/nm^2^ (red circles) and σ = 0.042 chains/nm^2^ (green triangles), while for the previous brush, σ = 0.45 chains/nm^2^. Also, the brush studied here contains much longer chains (39 kDa) than previously (=2 kDa). Both the sparser grafting and longer chain length result in chains that can be stretched much more easily than those studied previously [16]. For the more extensible chains studied here, it can be seen that the dependence on salt concentration, in the OB regime, shows a power-law exponent of around ~+0.13 to +0.15, and in the SB regime, a ~−0.017 to −0.20 power-law exponent is seen (see log-log fit slopes for σ = 0.023 chains/nm^2^ (red circles) and σ = 0.042 chains/nm^2^ (green triangles)). Although from Figure 1 it may appear that the height is relatively independent of grafting density in the OB regime below the maximum brush thickness at 10 mM KCl, it is apparent that at salt concentrations of 7–10 mM, there is a slight decrease of brush height with increased grafting density. This could be indicative of the OB regime with a weakly negative scaling dependency predicted by Zhulina et al. [17]. However, we note the limitations of our system: the PAA brushes investigated here, while more elastic than those studied previously [16], still possess finite extensibility which likely suppresses or weakens the inverse scaling of brush height with grafting density. We note that shorter chains, which are even more subject to the effects of finite extensibility, show a monotonic increase in brush height with grafting density at fixed salt concentration in the osmotic brush regime [1]. Thus, it appears that a PAA chain of 39 kDa is close to the minimum chain length capable of showing an inverse dependence of brush height on grafting density, and that the OB scaling $H\sim \sigma^{-\frac{1}{3}}$ with exponent −1/3, of Zhulina et al., perhaps only applies for much longer chains than this.

### 3.3. Hysteresis Studies

**Figure 2:**
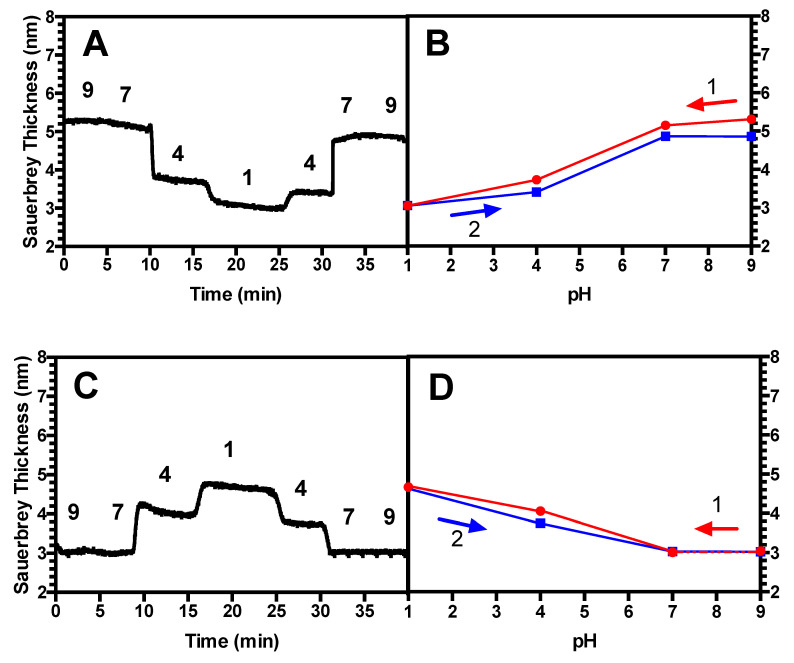
(**A**) Representative thickness responses to pH changes for a 2 kDa PAA brush with no polyethyleneimines (PEI) adsorbed, grafting density σ = 0.87 in 100 mM KCl with pH varied as shown, with effect of overlying solvent subtracted, as discussed at the end of the methods section. (**B**) Time-averaged thickness response vs. pH obtained from (**A**), with numbers indicating the order in which the pH scans occurred. Error bars in (**B**) and (**D**) represent the standard deviation for N = 3, but are invisible since they are smaller than the symbols. (**C**) Representative thickness response for a (PAA/PEI) bilayer with a 2 kDa PAA brush underlayer, grafting density σ = 0.87 in 100 mM KCl and 2.5 kDa PEI adsorbed overlayer, with solvent subtracted. (**D**) Time-averaged thickness response vs. pH obtained from (**C**). The dashed line represents a connecting segment that falls on top of another segment.

Figure 2A shows a time-dependent swelling response, or Sauerbrey thickness in nm, of a short (2 kDa) PAA brush with a grafting density σ of 0.87 chains/nm^2^ as pH is varied in steps, at fixed salt concentration. As in other figures below, the results shown are from one of the three replicates of the same experiment, with all three showing nearly identical results in all cases, as shown by the small standard deviations given in the averaged results in Figure 2b. This brush should be considered to be “dense” [33], in that the chains are spaced at distances much closer than the free chain’s radius of gyration, putting them in the “brush” rather than “mushroom” regime. In our previous work, it was demonstrated that this brush is quite extended, exhibiting a swelling/deswelling profile that disagrees with scaling theory for brushes that are low-to-moderately densely grafted [16]. The brushes collapse when the pH is decreased in agreement with what was seen by others including Yadav et al. [19], presumably due to the protonation of the chains, leading to the expulsion of salt counterions, and accompanying water, from the brush. Conversely, the brushes swell when the pH is increased, in agreement with others [19], as the chains are ionized, and salt ions and water then enter the brush. It can be seen in Figure 2A that in a pair of pH sweeps the first of which drives PAA in three steps from ionized to neutral, and the second of which drives it back to ionized, the Sauerbrey thicknesses in the first sweep are recovered in reverse order, as expected, in the second. In Figure 2B, each of these average thicknesses (which are themselves averages over time—see the materials and methods section), is averaged across three triplicate runs, giving the average brush thickness versus pH with error bars representing the standard deviations. Averaged over three samples, there is minimal hysteresis, and the brush’s swelling history is found to be highly reproducible.

Next, we exposed the PAA brush to PEI of similar chain length to form a (PAA/PEI) bilayer. It can be seen in Figure 2C that this bilayer, when subjected to identical pH downward/upward sweeps, also recovers its original Sauerbrey thicknesses during the second, upward, sweep in pH. Unlike the brush, which shrinks as pH is reduced, notably, the bilayer swells as pH is lowered. LbL multilayers have been generally found to swell at reduced pH, as shown for example by Hiller and Rubner [34] for a multilayer of poly(allylamine) and poly(styrene sulfonate) and by Silva et al. [35] for an LbL multilayer of chitosan/alginate. The reason for this is unclear; however, it is hypothesized that this is potentially due to the incorporation of the polycation PEI, which in theory should possess the opposite swelling response to pH changes as that of a polyanion. Figure 2D demonstrates that this bilayer, averaged over three samples, also lacks hysteresis and the bilayer’s history is reproducible.

**Figure 3:**
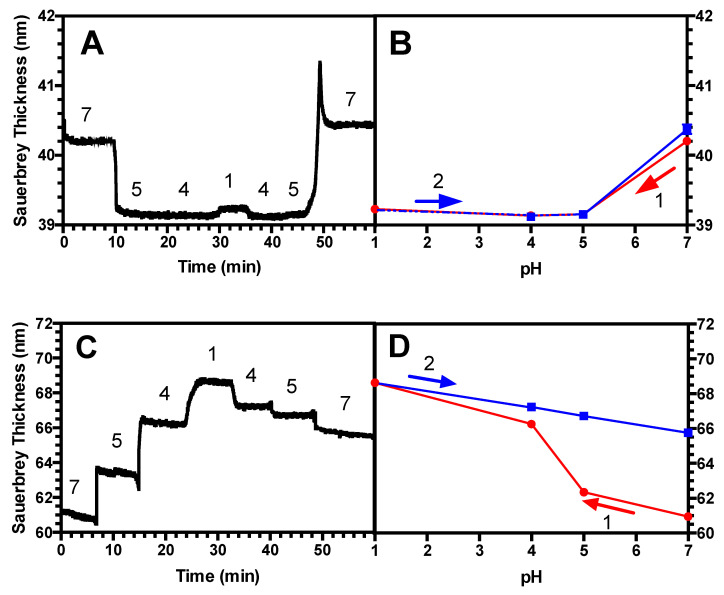
The same as in Figure 1 except for a 39 kDa PAA brush, grafting density σ = 0.45, again in 100 mM KCl; (**A**,**B**) are otherwise the same as in Figure 1, and in (**C**,**D**) the overlayer is a 25 kDa PEI. Error bars represent the standard deviation for N = 3 and if they are not visible, they are smaller than the symbols. The dashed line represents a connecting segment that falls on top of another segment.

Next, we studied a long-chain (39 kDa) PAA brush at a grafting density σ of 0.45 chains/nm^2^. This is considered to be “high” for this chain length, since in previous work, this brush was shown to be highly extended, similarly as the 2 kDa brush whose behavior is described in Figure 2A,B. Figure 3A shows that the height of this long-chain PAA brush decreases at low pH as was seen in the dense short brush of Figure 2A,B when subjected to a downward pH sweep, and it regains its original height when the pH is thereafter stepped upwards. Figure 3B shows that, averaged over three samples, there is negligible hysteresis, and the brush’s swelling history is highly reproducible. Then, we exposed this brush to PEI of similar chain length (25 kDa) to form a (PAA/PEI) bilayer, which was then subjected to an identical pH sweep. Figure 3C shows that the wet thickness at neutral pH = 7 of the bilayer is considerably greater than the thickness of the long brush alone, in Figure 3A, unlike what was seen for the short dense brush in Figure 2. This suggests that this long PAA brush is able to incorporate more PEI of similar length to that of the brush, than the short brush can. The dry thickness of this long brush is 27 nm, where the dry thickness of the bilayer is 34 nm. The long-chain bilayer’s response to the pH cyclic sweeps is found to be hysteretic, and it does not return to its original thickness when pH is increased again to pH 7. Figure 3D averages this time response across three measurements and shows good reproducibility, demonstrating that while a bilayer of dense PAA underlayer exhibits hysteretic swelling, a dense, monolayer PAA brush, does not.

**Figure 4:**
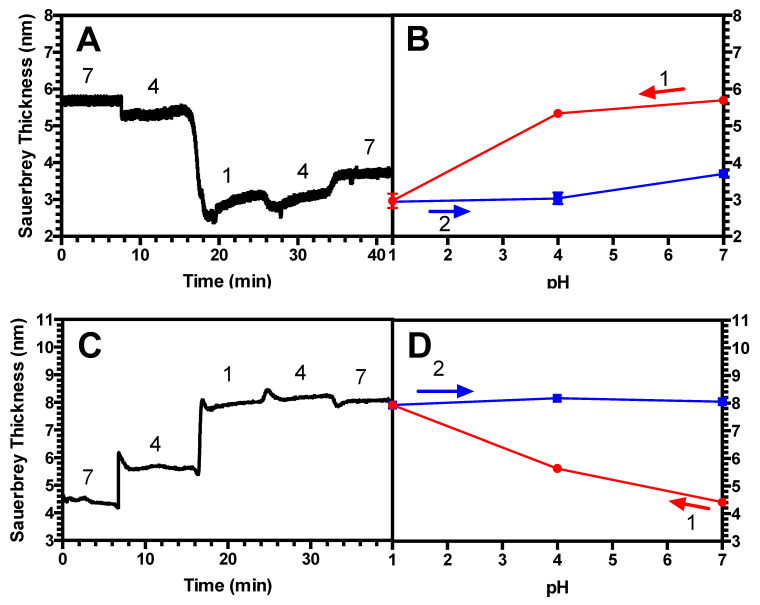
(**A**–**D**) are The same as in Figure 2, except for a grafting density σ = 0.023 in 100 mM KCl.

Next, we diluted this same long-chain (39 kDa) PAA brush to achieve a significantly less dense grafting density σ of 0.023 chains/nm^2^, with a dry thickness of 1.27 nm. This is considerably more dilute than the brush of grafting density σ = 0.45 chains/nm^2^ from Figure 3, which possessed a dry thickness of 27.0 nm. Figure 4A shows that the Sauerbrey thickness of this lower-density brush shrinks substantially in response to a decreasing pH. It should also be noted that the QCM-D data for this case do not reach as clear a steady state after each pH adjustment. This more sluggish response may be due to chains having more room to reconfigure themselves in response to pH changes than in the densely grafted brushes of Figure 2 and Figure 3. The values in Figure 4B, averaged over three measurements, demonstrate the hysteresis clearly. The error bars, representing the standard deviation of these three measurements, are visible above the size of the symbols, reflecting the greater variability of this measurement across replicate samples than for the measurements in Figure 2 and Figure 3. This is likely related to the lack of clear steady values at each pH in the sweep (see the materials and methods section for how the average values were obtained). After adsorbing PEI onto this brush, the PAA/PEI bilayer also swells hysteretically when subjected to a pH sweep, as shown in Figure 4C. However, the QCM-D time response shows less change over time after each pH change than does the monolayer brush. Nevertheless, when averaged over three measurements in Figure 4D, it can be seen that the hysteresis of these films is pronounced.

**Figure 5:**
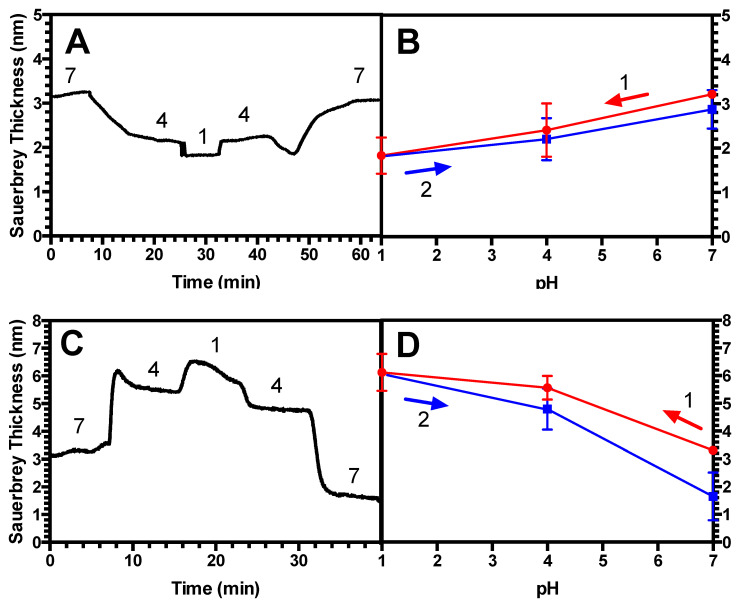
The same as Figure 2, except for a 14 kDa PAA brush; (**A**,**B**) are otherwise the same as in Figure 2, and in (**C**,**D**) 10 kDa PEI is used at grafting density σ = 0.06 in 100 mM KCl. Error bars represent the standard deviation for N = 3.

Thus, a dilute-grafted long-chain PAA brush and its corresponding (PAA/PEI) bilayer show hysteresis, while the densely grafted long-chain brush shows no measurable hysteresis, although its (PAA/PEI) bilayer does. To examine more precisely this transition to hysteretic behavior in long-chain, sparsely grafted brushes, we investigate a chain of intermediate length, 14 kDa, and grafting density, σ = 0.06 chains/nm^2^. Figure 5A shows that this brush, when subjected to a pH sweep, only slowly approaches a steady state, and as a result, only 3, rather than 4, different pH values were investigated. Despite the slowness of equilibration, Figure 5B shows that the brush de-swells with decreased pH as before, and when pH is increased again, does appear to approach its original height at each pH over an extended timescale. Figure 5B shows that, unlike the long-chain, dilute brush of Figure 3A,B, the intermediate-length brush at long times is not appreciably hysteretic; the error bars are comparable in size to the hysteretic gap in thicknesses. This medium-chain brush, when equilibrated with PEI of similar chain length to form a (PAA/PEI) bilayer, had a dry thickness of 1.70 nm compared to the 1.29 nm dry thickness of the PAA underlayer alone. In Figure 5C, it can be seen that the bilayer swelling is nearly, but not quite as, reversible when subjected to a reversing pH sweep, as the monolayer brush is, in Figure 5A. While the result in Figure 5D suggests some hysteresis, again, it is difficult to be sure due to the limited size of our sample (N = 3) and the magnitude of the error bars. Table 2 summarizes the hystereses for all PAA brushes and complementary bilayers.

**Figure 6:**
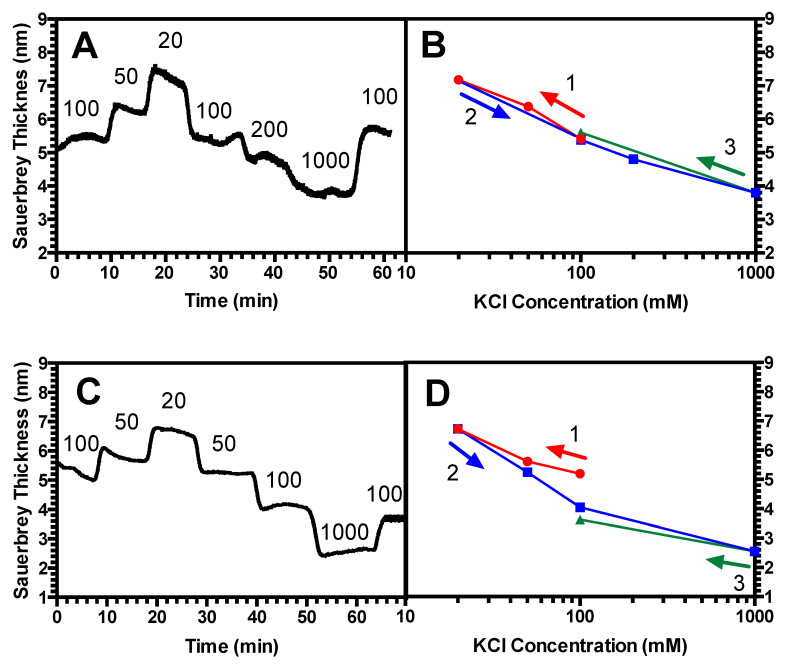
(**A**) Representative swelling response for a 2 kDa PAA brush, grafting density σ = 0.87 at fixed, neutral pH with varying KCl concentration in mM (values given in plots). (**B**) Average swelling vs. KCl concentration. (**C**) Representative swelling response for a 39 kDa PAA brush, grafting density σ = 0.023 at fixed, neutral, pH = 7 with varying KCl concentration in mM (values given in plots). (**D**) Average swelling vs. KCl concentration. Note that in (**C**,**D**), that the first and final salt concentration are the same and lie near the middle of the x axis at 100 mM.

Since in these studies, the salt concentration is fixed (at 100 mM) and pH is varied; as a control, it was also important to confirm the past experimental work where it was shown that LbL films did not exhibit hysteretic swelling/de-swelling when the salt concentration was changed at fixed pH [36]. As shown in Figure 6, we find that for a short, dense standalone PAA brush (chain length = 2 kDa, grafting density σ = 0.87) (Figure 6A,B), there is no measurable hysteresis; however, hysteresis is seen for a long-chain brush (chain length = 39 kDa) grafted at lower density than in our previous work (grafting density σ = 0.023) (Figure 6C,D). Both reversible and irreversible swelling at fixed pH and varied salt concentration have been seen in previous studies. For example, Sukhorukov and colleagues observed reversibility in swelling of multilayer films of poly(styrene sulfonate) (PSS) and poly(allylamine hydrochloride) (PAH) [36]. On the other hand, Cohen and coworkers have seen irreversibility which they attribute to rinsing off, or “etching” of the adsorbed polyelectrolyte in subsequent rinsing at high salt [37], a phenomenon which might also explain the hysteresis that we observe in Figure 6D.

Currently, no theory exists to predict the hysteresis of polyelectrolyte brushes or LbL films at fixed salt with changes in pH. We note that Yadav et al. found hysteresis with changes in pH in their materials that had polydispersity (M_w_/M_n_) of around 1.4, and found little hysteresis in their nearly monodisperse materials of similar average molecular weight. They therefore suggested that polydispersity is responsible for the hysteresis, but did not provide a mechanism for this. Here, we note that our materials possess dispersities of around 1.27, and if Yadav et al. are correct in their inference, the hysteresis in our materials may be related to their polydispersity.

## 4. Discussion

### 4.1. Scaling Theory for PE Brushes in the Osmotic Brush Regime

In this work, we revisited the major discrepancy between our previous experimental findings and the predictions of self-consistent field theory in the scaling of brush height with grafting density in the osmotic brush (OB) regime [17]. The theory of Zhulina et al. [17] predicts, seemingly paradoxically, that in the OB regime, a higher grafting density σ should lead to reduced brush height *H*, following the scaling law $H\sim \sigma^{-\frac{1}{3}}$ with exponent −1/3. Experiments in our previous work [16], and by Wu et al. [38], showed, instead, a positive scaling exponent, between 0.18 and 0.31, for the dependence of *H* on σ. The theoretical result was derived, however, for Gaussian chains, while our previous work and that of Wu et al. with PAA brushes measured the grafting density dependence for short brushes only, namely 2 kDa for our brushes and 4.10 kDa reported for the brushes of Wu et al. Because of their shortness, these brushes are expected to show non-linear (or non-Gaussian) elasticity. This may be at least partly responsible for the failure of the scaling law $H\sim \sigma^{-\frac{1}{3}}$ for weak polyelectrolytes in the OB regime. Using the longer 39 kDa chains and lower grafting density, in Figure 1, a decrease in σ no longer causes a decrease in brush height as seen for shorter brushes and denser grafting, but instead a slight increase in swelling with decreased σ in the OB regime near the peak brush height. This hints at a transition for longer brushes toward the scaling law $H\sim \sigma^{-\frac{1}{3}}$ predicted by Zhulina et al. for the OB regime. Future experimental work should examine the behavior of still longer brushes. The theory could also be improved by accounting for the finite extensibility to confirm that this suppresses the inverse dependence of brush height on grafting density. An interesting puzzle is that the OB scaling of brush height with the 1/3 power of ionic strength is observed even for short brushes, even though the prediction of the same theory for the dependence on grafting density fails, at least for the short brushes.

### 4.2. Hysteresis in Brushes and Bilayers

Next, we investigated the history-dependent swelling of a PAA brush as well as a (PAA/PEI) bilayer when pH or salt concentration is changed. We find that for a standalone PAA brush, when salt concentration is fixed, the brushes exhibit hysteresis in response to changes in pH when the chains are more elastic; namely, longer and less densely grafted (Figure 4A,B). By contrast, two less elastic brushes were found not to be hysteretic, including a short, and therefore more stretched, brush (Figure 2A,B), as well as a long-chain brush at a high grafting density (Figure 3A,B). For the (PAA/PEI) bilayer with a PAA brush underlayer, we demonstrated that the PAA underlayer plays a significant role in the swelling reversibility, leading to similar behavior as a standalone PAA brush. For a (PAA/PEI) bilayer with a short, more nearly fully extended underlayer, no measurable hysteresis is seen (Figure 2C,D), while for a PAA/PEI bilayer with a longer, more elastic brush underlayer, hysteresis is observed (Figure 4C,D). Hysteresis is also seen in the bilayer with a more densely grafted, longer-chain underlayer (Figure 3C,D). We also found that for moderate chain length and grafting density, both a standalone PAA brush and a (PAA/PEI) bilayer show hysteresis at short times, but for longer times, the brushes have less hysteresis (Figure 5). This work is the first experimental report of hysteresis in PAA and (PAA/PEI) brushes where the magnitude of the hysteresis is found to be a function of the brush properties. It is therefore a starting point for understanding the history dependence of the LbL process, which depends crucially on non-equilibrium transport, wherein polyelectrolyte deposition during one dipping step is not reversed during the rinsing or subsequent dipping steps. We note that in their studies, Yadav et al. found sensitivity of hysteresis to polyelectrolyte polydispersity, for polydispersities similar to those in our PAA (i.e., M_w_/M_n_ = 1.3 for 2 kDa; M_w_/M_n_ = 1.27 for 14 kDa, and M_w_/M_n_ = 1.4 for 39 kDa).

Intriguingly, we note that even an untethered polyelectrolyte in free solution, namely isotactic poly(methacrylic acid) (iPMA) in water, can show hysteresis in charge level upon change in pH [39]. Ghasemi and Larson [40] have suggested that this hysteresis is due to charge-conformation coupling in flexible weak polyelectrolytes. That is, a polyanion in a compact conformation resists deprotonation more strongly than does one in an extended conformation, because of the highly electrostatic repulsion in the former. Thus, a coiled chain will need a higher pH to achieve the same charge level than needed for an extended chain. Ghasemi and Larson suggested that this can lead to two conformation-charge states at the same pH, with at least one of the two being metastable. We hypothesize that, similarly, for tethered PAA chains, the resistance of monomers to deprotonation at a given pH depends on the chain stretch, producing two charge-conformation states at the same pH (at least one being metastable). If the energy barrier between these two states is high at intermediate pH values, then the pH history will determine which state is accessed, leading to hysteresis.

## 5. Conclusions

We have found a possible resolution to the one major discrepancy seen between previous measurements of brush height and scaling theory for weak polyelectrolyte in the osmotic brush regime. This discrepancy is the observed power-law increase in brush height H with increasing grafting density σ in contrast to the predicted scaling $H\sim \sigma^{-\frac{1}{3}}$. This discrepancy seems to be partially resolved by our observation that a longer PAA brush, 39 kDa, at lower grafting densities, namely σ = 0.023 and 0.042 chains/nm^2^, shows near the boundary between the osmotic brush and salted brush regime a slight *decrease* in brush height with increasing σ, in qualitative agreement with the scaling theory. This indicates that the scaling theory in the osmotic brush regime only fully applies for long enough brushes of sufficiently low grafting density. We also found that even a simple brush can show hysteresis in response to pH changes, especially if the brush is long or dilute. Hysteresis can also be seen in the corresponding bilayers when an overlayer of oppositely charged PEI is added to the brush. Hysteresis of this bilayer with respect to changes in salt concentration can also be observed, which may be due in part to rinsing away of some of the adsorbed PEI, similar to what is seen in some LbL multilayers during deposition. Thus, the behaviors of even simple brushes and bilayers have some of the complexity of LbL films, but provide much simpler systems whose theoretical analysis might in time lead aid in the analysis of the growth of LbL films.

## Figures and Tables

**Table 1:** Variable angle spectroscopic ellipsometry (VASE) results for the dry thicknesses of the poly(acrylic acid) (PAA) brushes and (PAA/PEI) bilayers investigated in this work.

Type of Material	Chain Length PAA/PEI (kDa)	PAA Brush Grafting Density, σ (Chains/nm^2^)	Dry Thickness (nm)
(PAA/PEI) Bilayer	2/2.5	0.87	2.80 nm ± 0.21
PAA brush only	2	0.87	2.66 nm ± 0.13
(PAA/PEI) Bilayer	14/10	0.060	1.70 nm ± 0.19
PAA brush only	14	0.060	1.29 nm ± 0.11
(PAA/PEI) Bilayer	39/25	0.45	34.3 nm ± 1.13
PAA brush only	39	0.45	27.0 nm ± 2.04
(PAA/PEI) Bilayer	39/25	0.023	2.02 nm ± 0.22
PAA brush only	39	0.023	1.27 nm ± 0.17
PAA brush only	39	0.042	2.32 nm ± 0.24

**Table 2:** Summary of experimental findings for PAA brushes and LbL bilayers (PAA/PEI) for various chain lengths and grafting densities. ** and the yellow color indicate intermediate cases where slow approach to equilibrium suggests that hysteresis would be present at short times (less than 5 min) but becomes small at longer times (tens of minutes).

Type of Material	PAA Brush Grafting Density, σ (Chains/nm^2^)	Chain Length (kDa) PAA, PEI	Hysteresis Observed
(PAA/PEI) Bilayer	0.87	2, 2.5	No
PAA brush only	0.87	2	No
(PAA/PEI) Bilayer	0.06	14, 10	**
PAA brush only	0.06	14	**
(PAA/PEI) Bilayer	0.45	39, 25	Yes
PAA brush only	0.45	39	No
(PAA/PEI) Bilayer	0.023	39, 25	Yes
PAA brush only	0.023	39	Yes

## Data Availability

The data presented in this study are openly available in http://cheresearch.engin.umich.edu/larson/research_polyelectrolytes.html (accessed on 5 March 2021).
